# Effect of Huangqin Tang on Urine Metabolic Profile in Rats with Ulcerative Colitis Based on UPLC-Q-Exactive Orbitrap MS

**DOI:** 10.1155/2020/1874065

**Published:** 2020-04-22

**Authors:** Dunfang Wang, Xuran Ma, Shanshan Guo, Yanli Wang, Tao Li, Dixin Zou, Hongxin Song, Weipeng Yang, Yongxiang Ge

**Affiliations:** ^1^Institute of Chinese Materia Medica, China Academy of Chinese Medical Sciences, Beijing 100700, China; ^2^The Experimental Research Center, China Academy of Chinese Medical Sciences, Beijing 100700, China; ^3^Beijing Hospital of Integrated Traditional Chinese and Western Medicine, Beijing 100039, China

## Abstract

As a classic prescription, Huangqin Tang (HQT) has been widely applied to treat ulcerative colitis (UC), although its pharmacological mechanisms are not clear. In this study, urine metabolomics was first analysed to explore the therapeutic mechanisms of HQT in UC rats induced by TNBS. We identified 28 potential biomarkers affected by HQT that might cause changes in urine metabolism in UC rats, mapped the network of metabolic pathways, and revealed how HQT affects metabolism of UC rats. The results showed that UC affects amino acid metabolism and biosynthesis of unsaturated fatty acids and impairs the tricarboxylic acid cycle (TCA cycle). UC induced inflammatory and gastrointestinal reactions by inhibiting the transport of fatty acids and disrupting amino acid metabolism. HQT plays key roles via regulating the level of biomarkers in the metabolism of amino acids, lipids, and so on, normalizing metabolic disorders. In addition, histopathology and other bioinformatics analysis further confirm that HQT altered UC rat physiology and pathology, ultimately affecting metabolic function of UC rats.

## 1. Introduction

Ulcerative colitis (UC) is a common inflammatory bowel disease, with abdominal pain, diarrhea, mucus, and pus, sustained or repeated as the main symptom [1]. The disease is considered to be a precancerous lesion of colon cancer, and it has been listed as one of the refractory diseases by the World Health Organization. It is widely believed that UC is the result of a combination of factors including genetics, gastrointestinal flora imbalance, and inflammatory overreaction [2–4]. Traditional Chinese medicine (TCM) is a complex and interactive system, which is frequently used in the form of formula (the combination of several different herbs) [5]. Compared with chemical treatment, TCM has the advantage of fewer adverse effects and is increasingly attracting researchers' attention for the treatment of gastrointestinal diseases [6]. Huangqin Tang (HQT), a well-known classic prescription for curing diarrhea, is a combination of four herbal medicines 3 : 2 : 2 : 2 by weight, namely, *Scutellaria baicalensis* Georgi, *Paeonia lactiflora* Pall, *Glycyrrhiza uralensis* Fisch, and *Ziziphus jujuba* Mill. Our previous work found that it has a good effect on intestinal mucosal inflammation in UC rat models [7, 8].

Metabolomics is defined as systematically qualitative and quantitative analysis of metabolites in a given organism or biological sample [9], which together with genomics, transcriptomics, and proteomics jointly constitutes the “Systems Biology” [10, 11]. As a systemic approach, metabolomics reflects the function of organisms from terminal symptoms of metabolic network by a “top-down” strategy and shows metabolic changes of a complete system caused by interventions in a holistic context [12, 13]. As an emerging field, LC-MS-based metabolomics has been frequently applied to the toxicity study of pure compounds, extracts, and compound prescriptions and is a good tool to evaluate toxicity of natural products systematically and explore the mechanisms of toxicities. Now, the focus of metabolic toxicity research is mainly on nephrotoxicity, hepatotoxicity, cardiotoxicity, and central nervous system toxicity [11].

UPLC-based metabonomics was also employed to analyse the key endogenous metabolites in the body fluids, which is now increasingly considered as a novel diagnostic approach in clinical studies including liver, lung, gastrointestinal, diabetes, urogenital, and other diseases. Simultaneously, novel and more sensitive biomarkers were found for early detection and diagnosis of these diseases [14–16]. The clinical application of metabonomics provides comprehensive information and improves the feasibility of high-throughput patient screening for diagnosis of disease status or risk evaluation. Most likely, identification of clinically relevant metabolites that may be regarded as potential new biomarkers will also help with the evaluation of prognosis and contribute to the development of new therapeutic strategies [17].

Through literature search, there are few reports on the metabolomics research of UPLC-MS combined with UC. Therefore, this study used metabolomics UPLC-MS technology to find biomarkers related to UC. At the same time, the effects of HQT on the metabolites of UC rats were analyzed, and the possible metabolic pathways in rats were revealed to further elucidate the mechanism of HQT in the treatment of UC.

## 2. Materials and Methods

### 2.1. Chemicals

2,4,6-Trinitrobenzenesulfonic acid was obtained from Sigma-Aldrich (St. Louis, MO, USA). IL-17 and PGE_2_ ELISA kits were purchased from Shanghai Panke Industrial Co., Ltd (Shanghai, China). The RNA PCR Kit was sourced from Takara (Dalian, China). Formic acid (LC-MS grade) was purchased from Tedia Company Inc. (Fairfield, OH, USA). Acetonitrile and methanol (LC-MS grade) were purchased from Sigma-Aldrich.

### 2.2. Animals

Wistar male rats (180–200 g) were obtained from the Laboratory Animal Center of the Academy of Military Medical Sciences (Production license no. SCXK 2012-0004). All rats were housed at 23 ± 1.5°C. Animal experiment process was conducted in accordance with the ethical guidelines for local animal care and usage.

### 2.3. Preparations of HQT

*Scutellaria baicalensis* Georg*i*, *Paeonia lactiflora* Pall, *Glycyrrhiza uralensis* Fisch, and *Ziziphus jujuba* Mill (weight ratio 3 : 2 : 2 : 3) were weighed and mixed. For the first decoction, the mixture was refluxed with water (1 : 10, w/v) for 1.5 h. The filtrates were collected, and then the water with residues (1 : 8, w/v) was boiled for an additional 1 h. Two batches of filtrates were gathered. Thereafter, the sample was dried under reduced pressure to obtain the HQT extract. Furthermore, the major active ingredients in the prescription were detected by using liquid chromatography-tandem mass spectrometer (LC-MS).

### 2.4. UC Rat Model Construction and Grouping

TNBS colitis in rats was induced according to previously reported methods [18]. 24 h fasted Wistar rats were moderately anesthetized with pentobarbital, and then carefully inserted a 0.56 mm catheter into the colon with the tip 8 cm proximal to the anus. To break the intestinal epithelial barrier and induce colitis, the mixed reagent containing 0.25 ml of 50% ethanol and TNBS (100 mg/kg) was slowly administered into the lumen by the catheter fitted onto a 1 ml syringe. Rats in the control group were all administered an equal volume of saline. Animals were kept in an upside-down position for 2 min and returned to their cages.

### 2.5. Serum Cytokine Detection

After a ten-day administration, blood samples were collected by the eyelid method and then centrifuged (3,000 rpm, 15  min) to get serum. Production of NO in serum was measured by Griess assay; the levels of proinflammatory cytokines IL-17 and PGE_2_ in serum were detected by using ELISA kits in accordance with the manufacturer's specifications.

### 2.6. Histological Examination

After the colonic segments were collected from all three rat groups, the tissue was embedded and sliced as usual. Then, 4 *μ*m-thick tissue sections were prepared and stained with hematoxylin and eosin (H&E) for histological studies. The extent of colonic lesions was compared based on the ulcer size, inflammatory infiltration, and structural damage.

### 2.7. Reverse Transcription-PCR

Colonic segments were frozen and ground in liquid nitrogen. Then, 2 *μ*g RNA was used for reverse transcription. Primers for FABP1 and FABP2 and GAPDH are given in Table 1. Moreover, the mixture was diluted 10-fold, and final reaction volumes of 20 *μ*l were used for data analysis. Reactions were run in triplicate. The relative levels of FABP1 and FABP2 were calculated by the 2^−ΔΔCt^ method [19].

### 2.8. Urine Sample Collection

After the last administration, all rats were fasted for 24 hours, and then urine was collected the next morning and centrifuged for 10 min at 3000 rpm. Each urine sample (50 *μ*L) was added to 450 *μ*L acetonitrile agent. The samples were vortexed for 30 seconds and centrifuged at 13,000 rpm for 10 minutes at 4°C. Thereafter, 100 *µ*L supernatant was transferred to an autosampler vial for analysis with a UPLC-Q-Exactive hybrid quadrupole-Orbitrap mass spectrometer. The mobile phases included A (0.1% formic acid and 2 mmol/L ammonium formate in water) and B (acetonitrile). The gradient procedure was as follows: 0～1 min, 95～95% A; 1～15 min, 95%～5% A; 15～16 min, 5%～5% A; 16～17 min, 5%～95% A; and 17～18 min, 95%～95% A. The analysis time of the sample is 0 to 18 minutes. The ion source optimal conditions were set as follows: evaporation temperature, 350°C; sheath gas, 35 Arb; spray voltage, 2.8 kV; auxiliary gas, 10 Arb; capillary temperature, 320°C; and S-lens RF, 50. The compound parameters were set as follows: grade I, full scan (±); resolution, 70,000; maximum TT, 100 ms; and AGC target, 1e^6^; m/z, 70–1050.

### 2.9. Statistical Analysis

The mzCloud (Thermo Fisher Scientific) was used to obtain the exact mass [20]. Then, we used TraceFinder software (Thermo Fisher Scientific) to qualitatively analyze the peak area of each endogenous metabolite in the sample. Next, identified ions were confirmed by databases including Metlin (http://metlin.scripps.edu/), the Human Metabolome Database (http://www.hmdb.ca) [21], and the Kyoto Encyclopedia of Genes and Genomes (http://www.genome.jp/kegg/ligand.html) [22, 23]. Principal component analysis (PCA) and partial least squares-discriminant analysis (PLS-DA) were performed using MetaboAnalyst 4.0 to identify discriminant metabolites. The data were statistically processed using SPSS 11.0 (SPSS Inc, Chicago, IL, USA). Our data were expressed as mean ± standard deviation (SD), and Student's *t*-test or one-way ANOVA was used for comparison between groups.

### 2.10. Metabolic Pathways Analysis

Pathway analysis of potential biomarkers (VIP values >1) was assessed using MBRole. The critical *p* value obtained from pathway analysis was set as <0.01. Then, we used the Metscape model, which was utilized to visualize interactions among compounds, enzymes, and genes in Cytoscape, to map a functional network of the major endogenous metabolites related to UC.

## 3. Results

### 3.1. Chemical Compounds of HQT

LC-MS was performed to identify the major active ingredients in HQT. The 11 main active-ingredient chemical compositions were as follows: baicalin (10.4149%), wogonoside (2.4179%), oroxylin-A-glucoside (0.8229%), baicalein (0.6754%), wogonin (0.2215%), oroxylin-A (0.0967%), liquiritin (0.0384%), isoliquiritin apioside (0.0354%), liquiritigenin (0.0280%), isoliquiritoside (0.0865 *μ*g/mL), and isoliquiritigein (0.0049%) [24].

### 3.2. HQT Promoted Recovery of the UC Rats

The UC rats induced by TNBS showed a series of symptoms within 3 days, such as apathetic, drowsiness, poor appetite, weight loss, diarrhea, and purulent stools. After prolonged HQT exposure, rats' response to abovementioned symptoms were all improved significantly. Their appetite was stimulated with decreased diarrhea. The model group rats had no obvious improvement in symptoms.

### 3.3. HQT Decreases Serum Level of Inflammatory Cytokines

**Figure 1:**
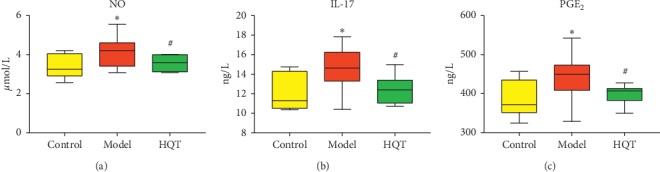
Effects of Huangqin Tang (HQT) on concentrations of (a) NO, (b) IL-17, and (c) PGE_2_ after 10 days of oral administration on ulcerative colitis rats. (*n* = 10, $\left(\bar{x}\right)\pm s$) ^*∗*^*P* < 0.05,^*∗∗*^*P* < 0.01 vs normal control; ^#^*P* < 0.05,  ^##^*P* < 0.01 vs model control.

The levels of proinflammatory factors NO, IL-17, and PGE_2_ in the model group were increased remarkably. In addition, levels of NO, IL-17, and PGE_2_ in the HQT group were clearly lower than those in the model group (Figure 1).

### 3.4. Histological Study

**Figure 2:**
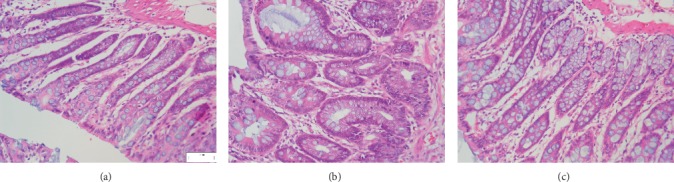
Histological observations of the colon tissues in different groups (H&E staining) (10 × 40). (a) Normal control; (b) UC Model control; (c) HQT 20 g·kg^−1^.

The rats with TNBS-induced colitis in the mucosa of colons revealed inflammatory cell infiltration, loss or enlargement of goblet cells and epithelium, and edema. Part of the colon gland disappeared. The histologic sections of the HQT group showed progressive restoration, improvement of intestinal ulcer, and reduction in infiltration of inflammatory cells and edema compared to the UC group (Figure 2).

### 3.5. The mRNA Levels of FABP1 and FABP2

**Figure 3:**
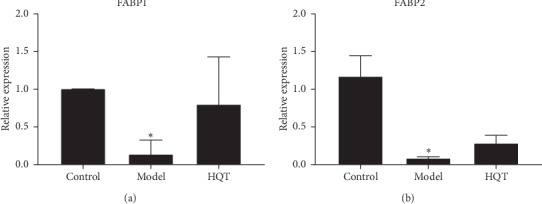
Effect of HQT on mRNA expression levels of the corresponding genes (a) FABP1 and (b) FABP2 according to real-time PCR analysis. Data are represented as mean ± S.E. “^*∗*^”, “^*∗∗*^”and *P* < 0.05, *P* < 0.01, respectively, as compared with the normal control group. “^#^” and “^##^”, *P* < 0.05 and *P* < 0.01, respectively, as compared with the model group.

The results of real-time PCR analyses indicated that the mRNA levels of FABP1 (*P* < 0.05) and FABP2 (*P* < 0.01) in colon tissue of UC model rats were significantly lower than those of normal rats but could be effectively increased by HQT treatment (Figure 3).

### 3.6. Diversified Analysis of Pattern Recognition of Urine Data

**Figure 4:**
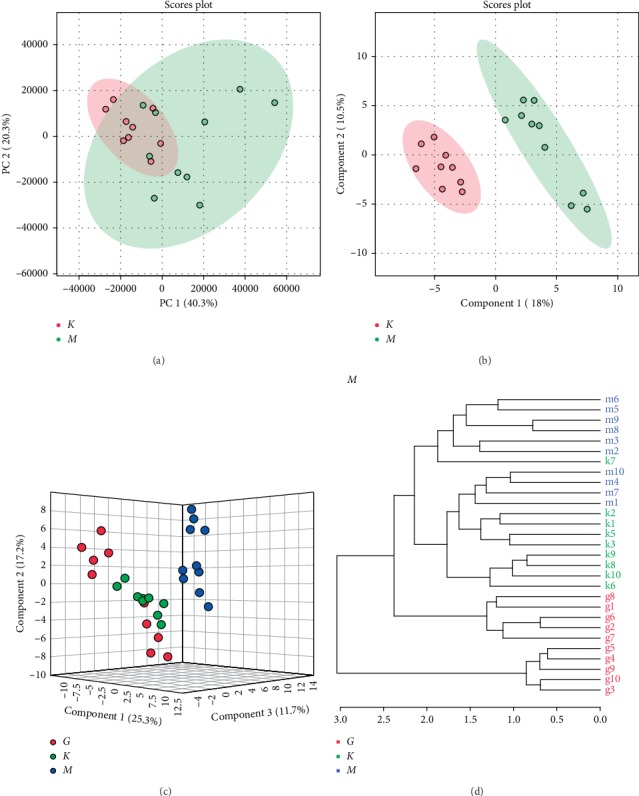
Metabolomics analysis of urine samples from control, model, and HQT rats (*K* denotes the control group, *M* denotes the model group, and *G* denotes the HQT group). (a) PCA score plots for comprehensive metabolomic data of the model and control groups. (b) PLS-DA score plots for comprehensive metabolomic data of the model and control groups. (c) Score plots of the OPLS-DA of the model, control, and HQT groups. (d) Cluster analysis was performed on the model group, control group, and HQT group (distance measurement method: euclidean; clustering method: ward. D).

Urine data from the control group and the model group were analyzed using unsupervised PCA. It can be seen from the scores in Figure 4(a) that there is a certain tendency for each group of samples to aggregate. In order to obtain more ideal intergroup separation and enhance the identification of variables that contribute to the classification, a supervised PLS-DA analysis was further carried out. As shown in Figure 4(b), the model group is clearly separated from the normal group, and the normal physiological metabolism of the rat body was disturbed. From the perspective of the change of physiological metabolites, the UC model can be considered to be successful. In Figure 4(c), there is no overlap between the normal group, the model group, and the HQT group. The HQT group is gradually closer to the normal group than the model group. It indicated that the endogenous metabolic pattern of the urinary part of UC rats changed after HQT administration, which indicated that HQT had a certain effect on regulating metabolic abnormalities in UC rats. The quality parameters of UC models were accuracy, 0.8947; *R*^2^, 0.8255; and *Q*^2^, 0.6065.

### 3.7. Identification of Potential Biomarkers

In the PLS-DA model, 28 variables (VIP >1.0) were identified as metabolic markers, as shown in Tables 2 and 3. Compared with the normal group, in the model group, L-isoleucine, pyroglutamic acid, L-histidine, 4-hydroxyproline, 3-methylhistidine, gamma-aminobutyric acid, L-valine, L-proline, L-leucine, glycine, m-methylhippuric acid, 5-hydroxyindoleacetic acid, 1,2,3-propanetricarboxylic acid, ethanolamine, adipic acid, and 4-hydroxyphenylacetic acid were significantly elevated; arachidonic acid, palmitoleic acid, alpha-linolenic acid, and oleic acid were significantly reduced. After the intervention of HQT, the abovementioned multiple differential metabolites have a tendency to approach normal levels, indicating that HQT has a regulating effect on the abovementioned metabolites. However, adipic acid, 4-hydroxyproline, etc., did not approach the normal value after drug treatment. The reasons for consideration may be as follows: on the one hand, the treatment time is still short; on the other hand, the disease changes still need a certain process.

### 3.8. Correlation and Cluster Analysis of Latent Biomarkers

**Figure 5:**
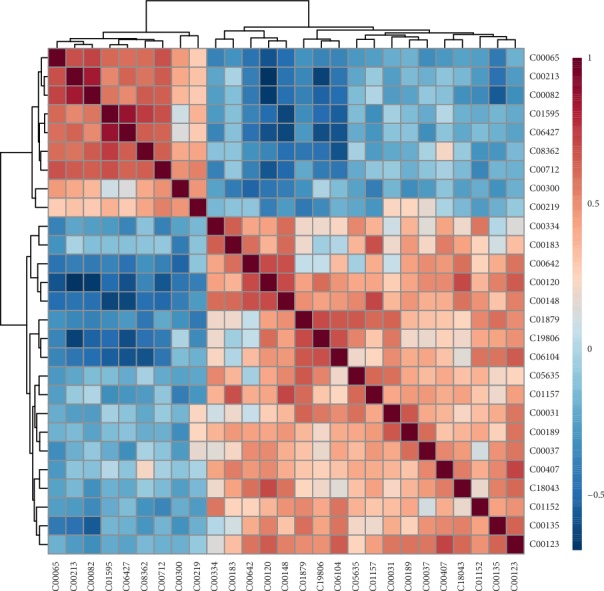
The correlation analysis of potential molecular markers in the UC model compared to the normal group.

The correlations between the trends of potential molecular markers are shown in the form of heat maps (Figure 5). The horizontal and vertical axes represent the variable information of endogenous substances, the light color indicates weak correlations, and dark color reflects strong correlations. Blue denotes a negative correlation and red indicates a positive correlation. The closer the correlation value is to 1, the more similar the trend between the two molecules is. Conversely, the correlation is less than zero, indicating that the change trend between the two molecular markers is opposite. As shown in Figure 5, amino acids and organic acids accumulate in an elevated trend in the model group, while fatty acids accumulate in a decreasing trend.

### 3.9. Exploratory Analysis of Diversified ROC Curves

**Figure 6:**
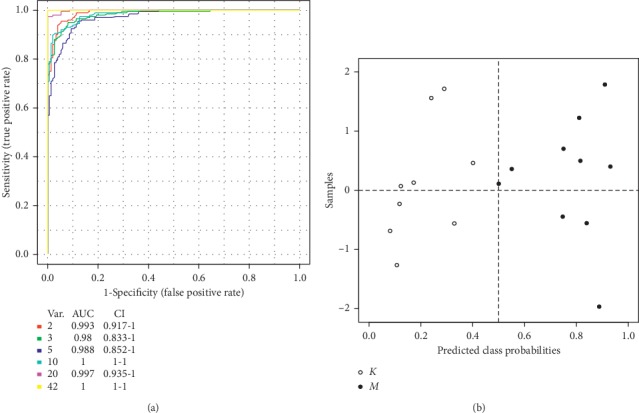
Comparison of the control and model groups of urine samples from multiple ROC curve analysis.

Screening for the clinical diagnosis of potential urinary biomarkers metabolomics is an important task, and the use of ROC curves to evaluate the diagnostic accuracy of biomarkers has been successful in many studies [25, 26]. AUC between 0.9 and 0.7 represents the diagnostic accuracy of a biomarker. Biomarkers with AUC values greater than 0.9 are highly accurate. As shown in Figure 6, the AUC value is >0.9, and the diagnostic sensitivity and specificity are high, indicating that metabolites used as markers for disease diagnosis have high clinical diagnostic value.

### 3.10. Metabolic Pathways Analysis

**Figure 7:**
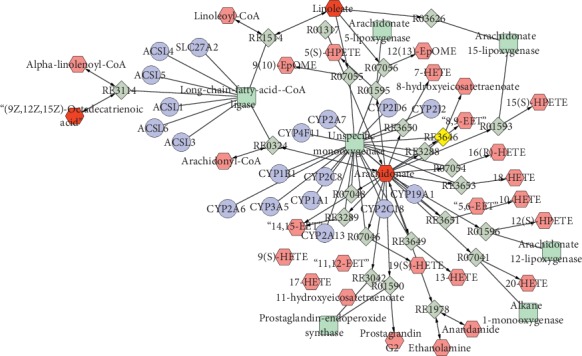
Compound-reaction-enzyme-gene network analysis of major endogenous metabolites in testis.

**Figure 8:**
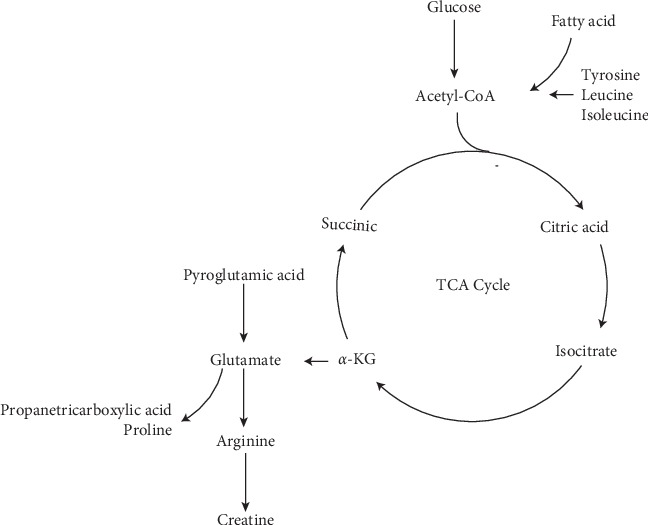
Metabolic profiles of potentially different metabolites in urine.

A total of 8 metabolic pathways (*p* value <0.01) related to UC impacted in the urine. These pathways included valine, leucine, and isoleucine biosynthesis, biosynthesis of unsaturated fatty acids, arginine and proline metabolism, and ABC transporters (Table 4). The abovementioned pathways may be the latent mechanisms underlying the changes of endogenous substance metabolism after 10 days of HQT exposure. Furthermore, several major metabolic disorders are connected in series, showing that UC is a disease involving multiple metabolic disorders (Figures 7 and 8).

## 4. Discussion

This study attempts to clarify the complex pathophysiological process from a metabolic perspective with the UPLC-Q-Exactive Orbitrap MS high-sensitivity detection platform. The monitoring of these differential metabolites is helpful to further understand the mechanism of action of HQT on UC. The following is a discussion of the biological significance of the molecular markers of the abovementioned potential molecules.

### 4.1. Effects of HQT on the Amino Acid Metabolism in UC Rats

Amino acid is the basic functional unit that constitutes protein molecules and is one of the essential nutrients for the human body. In the UC model group, the amino acid content was generally increased. Some scholars suggested that the body can repair the damaged mucosa by decomposing proteins, causing amino acid accumulation [27]. Leucine and isoleucine promote the body's anabolism (muscle growth) in a special way, relieve muscle breakdown, and play an important role in the repair of skeletal muscle microdamage [28]. Chinese herbalists believe that diarrhea manifests itself as the loss of transportation, lack of subtle substances, no muscle growth, fatigue, weight loss, etc. These symptoms are associated with excessive loss of isoleucine, valine, and leucine in the urine, which may lead to lack of nutrition.

Recently, studies have suggested that histidine and glycine have antioxidation and anti-inflammatory effects. They have been used in the treatment of diseases such as peptic ulcer, anemia, and allergies. For the treatment of inflammation of the gastrointestinal tract, histidine can exert anti-inflammatory functions and remove toxins, thereby enhancing the body's antioxidant capacity [29].

Gamma-aminobutyric acid (GABA), an active amino acid, is an important inhibitory neurotransmitter in the gastrointestinal nervous system [30]. GABA regulates the central nervous system, promotes cerebral cortical function and autonomic nerve function recovery, and enhances gastrointestinal motility [31]. GABA content in the model group was significantly increased, indicating abnormalities in the gastrointestinal nervous system of the rats. This may also be the main cause of gastrointestinal dysfunction of UC rats. After the administration of HQT, the GABA content was significantly increased. HQT may improve gastrointestinal tract sensitivity by regulating GABA, thereby improving diarrhea.

### 4.2. Effects of HQT on the Fatty Acid Synthesis and FABPs in UC Rat

Linoleic acid is a functional polyunsaturated fatty acid that enhances the body's immune function. It is mainly used to increase the production of nonspecific antibodies, promote lymphocyte proliferation, and enhance the phagocytic ability of phagocytic cells. Linoleic acid can increase the secretion of IgG, IgA, and IgM from rat intestinal lymph nodes and spleen lymphocytes, reducing the release of inflammatory cytokines [32]. The results of this study showed that the level of linoleic acid in the urine of the model group was significantly decreased, and the levels of inflammatory factors IL-17, PGE_2_, and NO in serum were increased, suggesting that the increase of inflammatory factors may be related to the decrease of linoleic acid level.

Simultaneously, Figure 7 displays that arachidonic acid levels were highly correlated with long-chain acyl-CoA ligase expression. The decreasing arachidonic acid may influence fatty acid synthesis, consistent with the metabolic pathway analysis. In the urine, the levels of linoleic acid, *α*-linolenic acid, and oleic acid, closely related to the activity of long-chain fatty acyl-CoA synthetase, also showed decreasing trends. The result demonstrated that fatty acid synthesis was disturbed by UC. In addition, the transport of fatty acids is believed to be performed by the FABP family, and FABPs activation can increase uptake of fatty acids [33]. The result showed that FABP1 and FABP2 were downregulated in the inflamed UC mucosa. It indicates that the genes of FABP1 and FABP2 were inhibited, affecting fatty acid uptake. This also confirms the metabolic pathway of ABC transporters. After 10 days of exposure to HQT, arachidonic acid, linoleic acid, *α*-linolenic acid, oleic acid, FABP1, and FABP2 were upregulated, indicating HQT profoundly regulates the disrupted fatty acid synthesis and metabolism.

### 4.3. Effects of HQT on the TCA Cycle and Energy Supply in UC Rat

Glucose, pyruvic acid, and fumaric acid are important endogenous substances for the TCA cycle. The proportion of pyruvic acid and fumaric acid was lower than that in the normal rat, and the proportion of glucose and lactic acid were higher than that in the control rat (Tables 3 and 5). The results illustrated that it might have been caused by the impaired TCA cycle. The increasing trend of lactic acid in UC may validate the hypothesis that the rat obtains energy from anaerobic glycolysis and thus produces lactic acid even under aerobic conditions [34].

Creatine provides energy to muscles and nerve cells. It is a nitrogen-containing organic acid that naturally occurs in vertebrates [35]. Due to the small amount of ATP stored in the body, the existing ATP will soon be consumed during exercise. Creatine can quickly synthesize ATP to provide energy to meet the body's movements [36]. Roediger [37] believes that UC is an energy-deficiency disease. A large amount of ATP was depleted in the UC rat, causing creatine to rapidly synthesize ATP to provide energy, which may be the main reason for the decline of creatine level in UC rats. The observed decreased creatine level was also another manifestation of the insufficient ATP supply.

This study explored the mechanism of action of HQT in rats with UC from the perspective of metabolomics. It was found to act by regulating multiple metabolic pathways such as valine, leucine, and isoleucine biosynthesis, biosynthesis of unsaturated fatty acids, arginine and proline metabolism, and ABC transporters. It embodies the characteristics of multitarget and synergy of traditional Chinese medicine compounds. In addition to amino acids, lipids and other ingredients, cholesterol sulfate, 2-methyl hippuric acid, 4-hydroxyphenylacetic acid, and other ingredients will also affect the progression of UC.

The UC biomarkers obtained in this experiment have something in common with the clinic [38, 39]. Arginine and proline metabolism and glycine, serine, and threonine metabolism are their common pathways, which proves that the development of UC is related to the abovementioned metabolic pathways. In addition, HQT has different degrees of regulation on the abovementioned biomarkers. These biomarkers are mainly amino acids, neurotransmitters, and fatty acids. These biomarkers are similar to those associated with UC in humans. Therefore, we speculate that HQT may work by regulating the abovementioned metabolic pathways. The speculation for this part also needs to be verified in conjunction with target metabolomics studies. Secondly, proteomics, transcriptomics, and other biological experimental methods are used to explore the biological significance of these biomarkers in detail.

## Figures and Tables

**Table 1:** Primer used for the quantitative PCR.

Gene	Primer sequence 5′⟶3′
FABP1	F: 5′-TACCAAGTGCAGAGCCAAGAG-3′
	R: 5′-TGACCTTTTCCCCAGTCATGG-3′

FABP2	F: 5′-TGGGCATTAACGTGGTGAAGA-3′
	R: 5′-GTCCAGGTCCCAGTGAGTTC-3′

GAPDH	F: 5′-GAGTCAACGGATTTGGTCGT-3′
	R: 5′-GACAAGCTTCCCGTTCTCAG-3′

**Table 2:** Potential biomarkers of ulcerative colitis.

Metabolite	RT (min)	Actual mass	Formula	KEGG
m-Methylhippuric acid	3.70	193.1992	C_10_H_11_NO_3_	—
5-Hydroxyindoleacetic acid	3.47	191.1834	C_10_H_9_NO_3_	C05635
1,2,3-Propanetricarboxylic acid	0.99	176.1241	C_6_H_8_O_6_	C19806
Ethanolamine	1.21	61.0831	C_2_H_7_NO	C00189
Adipic acid	1.05	146.1412	C_6_H_10_O_4_	C06104
L-isoleucine	1.02	131.1729	C_6_H_13_NO_2_	C00407
Pyroglutamic acid	0.87	129.114	C_5_H_7_NO_3_	C01879
L-histidine	13.52	155.1546	C_6_H_9_N_3_O_2_	C00135
4-Hydroxyproline	0.93	131.1299	C_5_H_9_NO_3_	C01157
Sarcosine	0.87	89.0932	C_3_H_7_NO_2_	C00213
3-Methylhistidine	1.66	169.1811	C_7_H_11_N_3_O_2_	C01152
Gamma-aminobutyric acid	0.96	103.1198	C_4_H_9_NO_2_	C00334
Linoleic acid	9.48	280.4455	C_18_H_32_O_2_	C01595
L-valine	1.02	117.1463	C_5_H_11_NO_2_	C00183
Creatine	1.02	131.1332	C_4_H_9_N_3_O_2_	C00300
Cholesterol sulfate	8.43	466.717	C_27_H_46_O_4_S	C18043
Arachidonic acid	9.16	304.4669	C_20_H_32_O_2_	C00219
L-tyrosine	7.4	181.1885	C_9_H_11_NO_3_	C00082
Palmitoleic acid	9.5	254.4082	C_16_H_30_O_2_	C08362
Alpha-linolenic acid	9.44	278.4296	C_18_H_30_O_2_	C06427
Oleic acid	9.55	282.4614	C_18_H_34_O_2_	C00712
Biotin	3.61	244.311	C_10_H_16_N_2_O_3_S	C00120
L-proline	0.99	115.1305	C_5_H_9_NO_2_	C00148
L-leucine	1.35	131.1729	C_6_H_13_NO_2_	C00123
Glycine	3.72	75.0666	C_2_H_5_NO_2_	C00037
D-glucose	0.99	180.1559	C_6_H_12_O_6_	C00031
L-Serine	14.76	105.0926	C_3_H_7_NO_3_	C00065
4-Hydroxyphenylacetic acid	1.95	152.1473	C_8_H_8_O_3_	C00642

**Table 3:** The average changes of potential biomarkers (VIP >1) in the urine.

Metabolite	Control	Model	HQT
m-Methylhippuric acid	96.02 ± 14.06	171.94 ± 87.40^*∗*^↑	131.78 ± 65.45↓
5-Hydroxyindoleacetic acid	1.64 ± 0.36	3.30 ± 2.18^*∗*^↑	1.51 ± 0.67^#^↓
1,2,3-Propanetricarboxylic acid	3.49 ± 0.58	4.48 ± 0.91^*∗*^↑	4.33 ± 0.74↓
Ethanolamine	1.51 ± 0.39	2.03 ± 0.52^*∗*^↑	1.60 ± 0.29^#^↓
Adipic acid	2.56 ± 0.23	2.92 ± 0.39^*∗*^↑	4.83 ± 1.91
L-isoleucine	9.30 ± 1.92	11.27 ± 1.75^*∗*^↑	11.79 ± 0.31
Pyroglutamic acid	5.20 ± 1.43	7.98 ± 2.68^*∗*^↑	7.34 ± 2.28↓
L-histidine	0.16 ± 0.05	0.25 ± 0.07^*∗∗*^↑	0.16 ± 0.10^#^↓
4-Hydroxyproline	0.68 ± 0.11	0.96 ± 0.25^*∗∗*^↑	1.38 ± 0.33
Sarcosine	0.32 ± 0.04	0.20 ± 0.07^*∗∗*^↓	0.52 ± 0.16^##^↑
3-Methylhistidine	0.75 ± 0.11	1.01 ± 0.30^*∗*^↑	1.57 ± 0.54
Gamma-aminobutyric acid	33.97 ± 6.74	46.78 ± 12.31^*∗*^↑	32.65 ± 14.94^#^↓
Linoleic acid	5.57 ± 1.26	4.45 ± 0.42^*∗*^↓	6.54 ± 2.66^#^↑
L-valine	40.17 ± 11.32	82.31 ± 42.31^*∗*^↑	73.53 ± 28.53↓
Creatine	7.04 ± 1.94	5.06 ± 0.99^*∗*^↓	6.05 ± 0.98^#^↑
Cholesterol sulfate	0.15 ± 0.02	0.24 ± 0.07^*∗∗*^↑	0.22 ± 0.09↓
Arachidonic acid	0.57 ± 0.17	0.39 ± 0.24^*∗*^↓	0.60 ± 0.18^#^↑
L-tyrosine	0.69 ± 0.12	0.41 ± 0.17^*∗∗*^↓	0.68 ± 0.25^#^↑
Palmitoleic acid	5.35 ± 0.82	4.47 ± 0.78^*∗*^↓	6.36 ± 2.23^#^↑
Alpha-linolenic acid	5.80 ± 1.05	4.64 ± 0.55^*∗*^↓	6.54 ± 2.66↑
Oleic acid	6.87 ± 1.68	5.06 ± 1.24^*∗*^↓	6.73 ± 1.99^#^↑
Biotin	1.44 ± 0.05	1.85 ± 0.13^*∗∗*^↑	2.27 ± 1.01
L-proline	0.14 ± 0.04	0.25 ± 0.03^*∗∗*^↑	0.35 ± 0.02
L-leucine	0.56 ± 0.07	0.71 ± 0.12^*∗∗*^↑	0.65 ± 0.21↓
Glycine	3.67 ± 0.34	5.83 ± 2.97^*∗*^↑	4.38 ± 2.45↓
D-glucose	3.87 ± 1.02	7.77 ± 7.72^*∗*^↑	6.28 ± 2.30↓
L-serine	11.85 ± 4.54	6.21 ± 2.04^*∗∗*^↓	9.01 ± 5.01↑
4-Hydroxyphenylacetic acid	23.13 ± 1.82	33.91 ± 6.71^*∗∗*^↑	31.50 ± 4.56↓

^*∗*^ *P* < 0.05, ^*∗∗*^*P* < 0.01 vs normal control; ^#^*P* < 0.05, ^##^*P* < 0.01 vs model group. Arrows indicate upregulated (↑) or downregulated (↓) metabolites in the model group compared with the control group and HQT group compared with model group.

**Table 4:** Result for urine pathway analysis with MBRole.

Pathway	Value	In set	%
Aminoacyl-tRNA biosynthesis	0.0000	8	32
ABC transporters	0.0000	8	32
Arginine and proline metabolism	0.0000	5	20
Glycine, serine, and threonine metabolism	0.0000	4	16
Biosynthesis of unsaturated fatty acids	0.0011	4	16
Valine, leucine, and isoleucine biosynthesis	0.0017	3	12
Cyanoamino acid metabolism	0.0051	3	12
Valine, leucine, and isoleucine degradation	0.0051	3	12

**Table 5:** Differential endogenous metabolites of UC rats.

Metabolite	Control	Model	HQT
Fumaric acid	6.75 ± 1.43	5.98 ± 0.93^*∗*^↓	6.93 ± 2.12↑
Pyruvic acid	7.34 ± 1.46	6.06 ± 0.74^*∗*^↓	7.17 ± 2.21↑
Lactic acid	16.11 ± 1.60	18.49 ± 2.78^*∗*^↑	11.30 ± 1.43^#^↑

^*∗*^ *P* < 0.05, ^*∗∗*^*P* < 0.01 vs normal control; ^#^*P* < 0.05, ^##^*P* < 0.01 vs model group. Arrows indicate upregulated (↑) or downregulated (↓) metabolites in the model group compared with the control group and HQT group compared with model group.

## Data Availability

The datasets used and analyzed during the current study are available from the corresponding author upon reasonable request.
